# Preferences for More or Less Health Care and Association With Health Literacy of Men Eligible for Prostate-Specific Antigen Screening in Australia

**DOI:** 10.1001/jamanetworkopen.2021.28380

**Published:** 2021-10-12

**Authors:** Kristen Pickles, Laura D. Scherer, Erin Cvejic, Jolyn Hersch, Alexandra Barratt, Kirsten J. McCaffery

**Affiliations:** 1 University of Sydney, Sydney Health Literacy Lab, School of Public Health, Faculty of Medicine and Health, Sydney, Australia; 2 Division of Cardiology, University of Colorado School of Medicine, Aurora; 3 VA Denver Center for Innovation, Denver, Colorado

## Abstract

**Importance:**

Understanding personal factors that influence diverse responses to health care information, such as preferences for more or less health care, might be beneficial to more effective communication and better involvement in health care choices.

**Objective:**

To determine whether individuals’ preferences for more or less health care are associated with informed choice and understanding of overdiagnosis in routine prostate cancer screening and to examine associations among preferences, educational status, and health literacy.

**Design, Setting, and Participants:**

This survey study included a community-based sample of men in Australia aged 45 to 60 years eligible for prostate-specific antigen (PSA) screening, recruited via an international social research company. Survey data were collected online from June 27 to July 26, 2018. Data were analyzed in April 2020.

**Exposures:**

Participants were randomized to 1 of 2 versions of an online decision aid (full-length or abbreviated) about PSA screening and completed an online survey that included a measure of preference for more or less health care, the Medical Maximizer-Minimizer Scale (MMS), in which higher score indicates preference for more health care.

**Main Outcomes and Measures:**

The primary outcome was informed choice; knowledge, attitudes, and intentions about screening for prostate cancer were also measured.

**Results:**

Of 3722 participants who began the survey, 2993 (80.4%) completed it (mean [SD] age, 52.15 [6.65] years). Stronger preferences for more heath care were observed in those without tertiary education (mean difference, 0.15; 95% CI, 0.09-0.22; *P* < .001) and with inadequate health literacy (mean difference, 0.16; 95% CI, 0.09-0.22; *P* < .001). After controlling for health and demographic variables, a 1-unit increase in MMS score was associated with reduced relative risk (RR) of making an informed choice (RR, 0.78; 95% CI, 0.74-0.82; *P* < .001) and of having adequate conceptual knowledge (RR, 0.87; 95% CI, 0.84-0.90; *P* < .001), correct numerical knowledge (RR, 0.93; 95% CI, 0.89-0.97; *P* = .001), and correct understanding of overdiagnosis (RR, 0.84; 95% CI, 0.79-0.90; *P* < .001). A 1-unit increase in MMS score was associated with a more positive attitude toward screening (RR, 1.18; 95% CI, 1.15-1.21; *P* < .001) and more positive intention to screen (RR, 1.20; 95% CI, 1.16-1.25; *P* < .001) after adjusting for control variables.

**Conclusions and Relevance:**

This survey study examined associations between preferences for more or less health care and knowledge about overdiagnosis and informed choice among men in Australia. These results may motivate clinicians to elicit individual patient preferences to facilitate tailored discussions with patients about low-value care, such as prostate cancer screening, for which benefit is uncertain.

## Key Points

**Question:**

What is the association of individuals’ preferences for more or less health care with knowledge about prostate cancer overdiagnosis and informed choice?

**Findings:**

In this survey study of 2993 men in Australia, higher scores on the Medical Maximizing-Minimizing Scale, indicating stronger preferences for more health care, were associated with reduced relative risk of making an informed choice, having adequate conceptual and correct numerical knowledge of prostate cancer screening outcomes, and correct understanding of overdiagnosis.

**Meaning:**

These findings may help clinicians to better tailor discussions with patients about low-value care, such as prostate cancer screening, for which benefit is uncertain.

## Teaser

This survey examines the association of preferences for more or less health care with informed choice among men in Australia eligible for prostate-specific antigen screening.

## Introduction

The Medical Maximizer-Minimizer theory proposes that people’s maximizing vs minimizing orientation is a stable trait that influences the way individuals use health care across time and contexts.^1^ Individuals with a maximizing orientation tend to prefer to take medical action when it comes to their health, even when there is significant burden of doing so and a low chance of benefit. By contrast, individuals with a minimizing orientation usually prefer to avoid medical tests and treatments, especially when there is significant treatment burden and a low chance of benefit. The Medical Maximizer-Minimizer Scale (MMS), a 10-item questionnaire developed and validated by Scherer and colleagues,^2^ assesses people’s preferences for more or less medical care and quantifies the orientation into a numerical score. Consistent with its theoretical underpinnings, the scale has been found to project health care utilization in a variety of contexts.^2^ People scoring highly on the scale (ie, people with a preference for more health care and medical intervention) have been shown to use more prescription medications^2^ and visit a physician more frequently,^3^ be more likely to get imaging tests^3^ and blood draws,^2^ and report more overnight hospital stays in the past 10 years^2^ compared with people with a preferences for less care. These associations are present even though individuals preferring more health care are typically no more ill than those preferring less and are equally likely to have health insurance.^3^

Routine population screening for prostate cancer using the prostate-specific antigen (PSA) test is not endorsed internationally, but it is widely used in clinical practice.^4,5^ Evolving evidence and guidelines continue to prompt substantial debate regarding the utility of PSA screening, particularly in higher-risk subgroups.^6^ The US Preventive Services Task Force (USPSTF) and Australian National Health and Medical Research Council (NHMRC) currently recommend a shared decision-making approach, whereby PSA screening should not occur unless an individual expresses a preference for screening after being informed of and understanding the benefits and harms.^7,8^ This is a shift from the USPSTF’s previous recommendation in 2012 that explicitly recommended against PSA screening in men of all ages. The major downside of PSA screening is the risk of overdiagnosis and overtreatment. Overdiagnosis occurs when a diagnosis is “correct” according to current professional standards, but the diagnosis or associated treatment has a low probability of benefiting the person.^9^ Cancer overdiagnosis refers to the detection of cancers that, in the absence of screening, would not present symptomatically.^10^

Some studies^11,12^ have suggested that guideline changes could have important implications for overdiagnosis; for example, PSA testing rates declined in the US following the 2012 USPSTF recommendation against PSA screening, which coincided with a decrease in early stage cancers detected, potentially diminishing rates of overdiagnosis. Associations of the 2018 USPSTF recommendation with screening behavior and patient outcomes will be informative to observe.

An informed choice is one that is based on relevant knowledge, consistent with the decision maker’s values, and behaviorally implemented.^13^ While the harms of overdiagnosis and overtreatment are particularly important to convey and understand if informed choice is to be achieved, they are also particularly difficult for physicians to explain and for patients to grasp.^14,15^ There is large variability in screening preferences and behavior among well-informed men.^16^

A 2020 study by Scherer et al^17^ found that MMS scores uniquely explained 29% of the variance in preferences for low benefit care. Previous studies have reported an association between MMS scores and men’s attitudes and intentions regarding prostate screening.^18^ In particular, another study by Scherer et al^18^ found that most men (both maximizers and minimizers) were initially interested in receiving PSA screening, but after being informed of the harms and low likelihood of benefit, men with a preference for more care remained enthusiastic about screening whereas men with a preference for less care became less likely to accept screening. This finding may indeed reflect good, preference-concordance choice. However, because knowledge about PSA screening was not assessed, the study was unable to examine whether maximizers and minimizers differed in terms of whether they made an informed choice. If maximizers (or minimizers) are ignoring evidence that is inconsistent with their preferences, then they might be making choices that are suboptimal because they are not well informed.

Our previous study involving almost 3000 men found that both long and abbreviated versions of a decision aid for prostate screening supported informed choice, and men in the longer decision aid group had significantly better understanding of overdiagnosis.^15^ The aim of this study is to investigate whether individuals’ preferences for more or less health care (measured using the MMS) were associated with informed choice and understanding of overdiagnosis in the context of PSA screening. Scherer et al^18^ have reported that individuals with higher levels of education tend to prefer less health care. In Australia, higher educated men with private health insurance are more likely to receive PSA tests than other demographic groups.^5^ We also explored associations between MMS scores and education and health literacy.

## Methods

This survey study was approved by the Human Research Ethics Committee of the University of Sydney. Completion and submission of the questionnaire was considered evidence of participant consent. This study followed the American Association for Public Opinion Research (AAPOR) reporting guideline.

We used a nonprobability internet panel and report participation rates. In 2018, an online randomized clinical trial was conducted in Australia comparing the effectiveness and acceptability of 2 versions of a decision aid (full length vs abbreviated) about prostate cancer screening.^15^ Findings of the decision aid trial have been published elsewhere.^15^ Participant preferences for more or less health care (assessed using the MMS) were measured in the baseline survey and are the focus of this analysis.

### Survey

The online survey was developed using internationally accepted, validated scales and items in previously published studies. The survey was built and administered using Qualtrics (SAP), an online survey platform.

Standard sociodemographic data were obtained from participants at baseline, including age, education, and private health insurance, as well as measures of health literacy adequacy^19^ and past experience with PSA testing. The MMS items were also included at baseline.

The MMS is a 10-item measure that assesses a person’s maximizing or minimizing tendencies (ie, preferences for more vs less health care) on a scale, from 1, indicating strongly disagree (strong minimizing) to 7, strongly agree (strong maximizing). MMS items capture the individual’s general health care preferences via their level of agreement or disagreement with statements such as “Doing everything to fight illness is always the right choice” and “It is important to treat disease even when it does not make a difference in quality of life.” The mean score for each participant is computed (score range, 1-7), with a higher score indicating a preference toward seeking health care at a greater frequency compared with those scoring lower on the scale.^2^ Generally, MMS scores are normally distributed around approximately the midpoint of the scale.^2,17,18,20^

After viewing the online decision aid, all participants responded to further questions in the online survey. These items were not assessed at baseline, only after the intervention, and included conceptual and numerical knowledge of prostate cancer, screening, and overdiagnosis; previous awareness of overdiagnosis; attitudes toward screening, diagnosis, and treatment; future screening intention; and perceived risk of developing prostate cancer. All survey items are presented in eTable 1 in the Supplement, and a detailed summary of the measures and analysis is reported elsewhere.^15^

The primary outcome was informed choice. It was based on the 3-dimensional framework by Marteau et al,^21^ which includes 3 constructs combining adequate knowledge of possible outcomes of screening and consistency between a respondent’s attitude toward the screening test (positive or negative) and intention to undergo a PSA test to determine the proportion of respondents who made an informed (or uninformed) choice.^21^

### Study Population and Data Collection

A community sample of men residing in Australia, aged 45 to 60 years, were recruited via an international survey sampling company (Survey Sampling International [SSI]) to participate in a 15-minute survey. SSI has an extensive online database panel of 600 000 members in Australia. Participants listed on the database have indicated a willingness to participate in online research in exchange for credits toward small rewards. SSI approached panel members who met this study’s eligibility criteria. Potential participants were emailed a web link to the online survey. All interested participants were directed to an online participant information statement; subsequent completion and submission of the questionnaire was considered evidence of consent. By virtue of being on the survey sampling database, all participants had already consented to being involved in online research.

All participants were recruited through the SSI database. SSI charges a set fee for providing the required number and type of survey participants. We requested quota sampling to ensure strong representation of men with lower educational attainment (ie, school-level qualifications only) and across the relevant age groups. Men who had previously been diagnosed with prostate cancer were excluded.

Survey data were collected from June 27 to July 26, 2018, through the Qualtrics platform. All men completed the same questionnaire.

### Statistical Analysis

Associations between MMS score and participant characteristics were explored using pairwise correlations and independent-sample *t* tests. Generalized linear models (with a Poisson distribution, log-link function, and robust SEs) were performed to obtain adjusted prevalence ratios (PRs) and relative risks (RRs) (with 95% CIs) per 1-unit increase in the MMS score (controlling for other variables, including decision aid group allocation) for making an informed choice, having a positive attitude toward PSA screening, previous awareness and correct understanding of overdiagnosis, having adequate knowledge of possible screening outcomes, and positive intention to screen. MMS score was treated as a continuous variable in all analyses. Analyses were conducted using Stata/IC statistical software version 15.1 (StataCorp). The threshold for statistical significance was set at 2-sided *P* < .05 for all analyses. Data were analyzed in April 2020.

## Results

### Participant Characteristics

Of 3722 participants who began the survey, 2993 men (80.4%) completed it and were included in analysis. Participants had a mean (SD) age of 52.15 (4.65) years, 1209 participants (40.4%) had no tertiary education, 1717 participants (57.4%) were privately insured, and most participants (2650 participants [88.5%]) had adequate health literacy (Table 1). The overall mean (SD) MMS score was 4.47 (0.90). The distribution of MMS score was approximately normal within the sample.

**Table 1.** Participant Characteristics

Characteristic	No. (%) (N = 2993)
Age, mean (SD), y	52.15 (4.65)
Education	
Tertiary	1784 (59.6)
No tertiary	1209 (40.4)
Private health insurance	1717 (57.4)
Previous PSA test	1155 (38.6)
Adequate health literacy	2650 (88.5)
Medical Maximizer-Minimizer Scale score, mean (SD)^a^	4.47 (0.90)

Abbreviation: PSA, prostate-specific antigen.

^a^ Range, 1 to 7, with higher scores indicating preference for more medical care.

### Univariable Unadjusted Analyses

There was no association between preferences for more or less health care and age (*r* = 0.027; 95% CI, −0.063 to 0.009; *P* = .14). A stronger preference for more care was observed in participants without tertiary education (mean [SD] MMS score, 4.56 [0.86]) compared with those with tertiary education (mean [SD] MMS score, 4.41 [0.92]; mean difference, 0.15; 95% CI, 0.09 to 0.22; *t*_2991_ = 4.56; *P* < .001; Cohen *d* = 0.17). Similarly, stronger preferences for more health care was reported by participants with inadequate health literacy (mean [SD] MMS score, 4.61 [0.84]) compared with those with adequate health literacy (mean [SD] MMS score, 4.45 [0.90]; mean difference, 0.16; 95% CI, 0.09 to 0.22; *t*_2991_ = 3.07; *P* < .001; Cohen *d* = 0.18).

Awareness and personal history of PSA testing were measured at baseline; 1155 participants (38.6%) reported a previous PSA test, and 1655 participants (55.3%) indicated that they had heard of the PSA test. For each 1-unit increase in MMS score, the prevalence of having heard about the PSA test significantly decreased by 9% (PR, 0.91; 95% CI, 0.88 to 0.94; χ^2^_1_ = 27.18; *P* < .001); yet there was no association between MMS score and previously having undergone a PSA test (PR, 0.99; 95% CI, 0.94 to 1.04; χ^2^_1_ = 0.22, *P* = .64).

Likelihood of perceived medium– to high–lifetime risk of prostate cancer (compared with no or low risk) increased by 8% per 1-unit increase in MMS score (RR, 1.08; 95% CI, 1.02 to 1.14; χ^2^_1_ = 7.85; *P* = .005). However, MMS scores were not associated with a difference in perceived risk of prostate cancer when participants were asked about their risk compared with the typical man of their age (RR, 1.07; 95% CI, 0.95 to 1.20; χ^2^_1_ = 1.22; *P* = .27).

### Multivariable Adjusted Analyses

#### Informed Choice

Having a preference for more health care was associated with lower rates of informed choice (Table 2). For each 1-unit increase in MMS score, the likelihood of making an informed choice was reduced by 22% (RR, 0.78; 95% CI, 0.74 to 0.82). The association between higher MMS score and reduced likelihood of making an informed choice was also maintained when previous awareness of overdiagnosis was added into the regression model (RR, 0.80; 95% CI, 0.76 to 0.84; χ^2^_1_ = 77.48; *P* < .001).

**Table 2.** Regression Models of Primary and Secondary Outcomes, With the RR and Associated Test Statistic and *P* value

Outcome	No. (%) (N = 2993)	RR (95% CI), per 1-unit increase in MMS		χ^2^_1_ Statistic	*P* value
		Unadjusted	Adjusted^a^		
Informed choice	1051 (35.1)	0.77 (0.73-0.81)	0.78 (0.74-0.82)^b^	92.58	<.001
Positive attitude toward screening	2239 (74.8)	1.18 (1.15-1.21)	1.18 (1.15-1.21)	168.5	<.001
Previous awareness of overdiagnosis	1563 (52.2)	0.82 (0.79-0.85)	0.83 (0.80-0.86)	100.8	<.001
Correct understanding of overdiagnosis	893 (29.9)	0.83 (0.78-0.88)	0.84 (0.79-0.90)	30.81	<.001
Correct conceptual knowledge	1777 (59.4)	0.86 (0.83-0.89)	0.87 (0.84-0.90)	73.5	<.001
Correct numerical knowledge	1351 (45.1)	0.92 (0.88-0.96)	0.93 (0.89-0.97)	10.73	.001
Positive screening intention	1676 (56)	1.19 (1.15-1.24)	1.20 (1.16-1.25)	109.9	<.001

Abbreviations: MMS, Medical Maximizer-Minimizer Scale; RR, relative risk.

^a^ All models adjusted for decision aid received, age, education, private health insurance, previous prostate-specific antigen screening, and health literacy adequacy.

^b^ This association was maintained when previous awareness of overdiagnosis was included in the regression model (RR, 0.80; 95% CI, 0.76-0.84; *P* < .001).

#### Attitude Toward Screening

After adjusting for the control variables, the MMS score was associated with attitude toward screening. The likelihood of having a positive attitude toward screening increased by 18% for each 1-unit increase in the MMS score (RR, 1.18; 95% CI, 1.15 to 1.21; χ^2^_1_ = 168.5; *P* < .001).

#### Awareness and Understanding of Overdiagnosis

After viewing the decision aid, participants were asked if they could recall seeing or hearing the term *overdiagnosis* before. Approximately half of participants (1563 participants [52.2%]) indicated that they had heard the term *overdiagnosis* before, but overall, understanding of overdiagnosis information was low.^12^ Regarding preferences for more or less care, after adjusting for control variables, the chance of having previous awareness of overdiagnosis reduced by 17% for each 1-unit increase in the MMS score (RR, 0.83, 95% CI, 0.80 to 0.86; χ^2^_1_ = 100.80; *P* < .001).

After adjusting for the same control variables, the chance of having a correct understanding of overdiagnosis was reduced by 16% for each 1-unit increase in the MMS score (RR, 0.84; 95% CI, 0.79 to 0.90; χ^2^_1_ = 30.81; *P* < .001). There was no association of greater understanding of overdiagnosis with health literacy adequacy (RR, 1.11; 95% CI, 0.91 to 1.35; χ^2^_1_ = 1.10; *P* = .29).

#### Conceptual and Numerical Knowledge

The chance of scoring correctly on conceptual knowledge items was reduced by 13% for each 1-unit increase in MMS score, after adjusting for all other control variables (RR, 0.87; 95% CI, 0.84 to 0.90; χ^2^_1_ = 73.54; *P* < .001). Individuals with adequate health literacy were more likely to respond correctly to conceptual knowledge questions compared with those with inadequate health literacy (RR, 1.31; 95% CI, 1.15 to 1.48; χ^2^_1_ = 17.45; *P* < .001).

After adjusting for other model variables, the likelihood of having correct numerical knowledge was reduced by 7% per 1-unit increase in MMS score (RR, 0.93; 95% CI, 0.89 to 0.97; χ^2^_1_ = 10.73; *P* = .001). There was no association of health literacy adequacy with numerical knowledge (RR, 1.11; 95% CI, 0.96 to 1.28; χ^2^_1_ = 2.01; *P* = .16).

#### Intention to Screen

After adjusting for all other model variables, the chance of having positive screening intention increased by 20% per 1-unit increase in MMS score (RR, 1.20; 95% CI, 1.16 to 1.25; χ^2^_1_ = 109.87; *P* < .001). Individuals with adequate health literacy were more likely to have positive screening intention compared with those with inadequate health literacy (RR, 1.19; 95% CI, 1.06 to 1.34; χ^2^_1_ = 9.14, *P* = .003).

#### Modification of Decision Aid Associations by MMS

The inclusion of an interaction term between MMS scores and decision aid received in the regression models found no difference in the association of decision aids with either informed choice (χ^2^_1_ = 0.26; *P* = .61) or intention to screen outcomes (χ^2^_1_ = 0.78; *P* = .38). Regression models were repeated post hoc to examine whether the association between MMS outcomes differed by whether or not a participant had previously had a PSA test, including an interaction terms between MMS score and previous PSA test (eTable 2, eFigure 1, and eFigure 2 in the Supplement).

## Discussion

This survey study contributes important new information to the understanding of medical maximizing-minimizing orientation, particularly regarding its association with knowledge-related outcomes. We found that individuals with a preference for more health care showed reduced understanding of overdiagnosis and less adequate knowledge about screening in general. MMS scores were associated with informed choice, ie, a choice that is based on relevant knowledge and shows consistency between the decision maker’s values and choice (in this case, operationalized as behavioral intention). Having a stronger preference for more care was associated with a lower likelihood of making an informed choice about screening for prostate cancer. Of particular note in this study was that maximizing was associated with greater risk of reduced understanding of overdiagnosis than were education and health literacy.

People’s innate preferences for more or less health care may guide what they direct their attention to and what they are willing to believe.^18^ Studies have shown that people will attend to and interpret information in a way that confirms their existing positions and ignore or dispute contradictory information.^22^ Such attentional and confirmatory biases can lead to heightened or reduced sensitivity to particular information. One possible explanation for our findings is that individuals with a strong preference for more health care gave less attention to information about screening harms, such as overdiagnosis, while those with a strong preference for less health care spent more time understanding and verifying reasons for not screening. Resultant knowledge possibly then influenced screening intentions. Unfortunately, timing data were not captured, so we are unable to evaluate whether men spent more or less time looking at particular information, such as overdiagnosis information, in the decision aids. Future research could capture timing data to test this hypothesis.

Alternatively, people who strongly prefer more health care might dismiss, reject, or disbelieve particular information about their preferred screening choice. They might not believe or accept the information about, for instance, the potential downsides of prostate screening or they may distrust the data presented. Women involved in focus groups about overdiagnosis of breast cancer often did not see information on overdiagnosis as relevant to their decision-making about screening, and most retained their initial (positive) perspectives on attending screening, perhaps reflecting the influence of broad and long-standing encouragement to be screened.^23^ A tendency to avoid or reject certain information in the context of health decisions is problematic because it can undermine engagement with potentially valuable risk information, such as overdiagnosis, that is perceived as threatening or unwanted.^24^

Differences in preferences for prostate screening were associated with differences in knowledge. However, we cannot say whether these differences were driven by an emotional motivation to decide to screen without attending to the information, or a failure to attend to and encode the information, or participants simply not believing or accepting it. Medical maximizing-minimizing orientation may influence screening intentions directly via the affect pathway (ie, via personal experiences of feeling or emotion). Affect can play multiple roles in medical decision-making,^25^ including influencing screening intentions.^26^ Men with an orientation toward more health care, for instance, might simply prefer to have a PSA screening test because of a “gut feeling” that it is the right thing to do in that moment.^27^ This theory may be partially supported by our data.

We found that MMS scores were lower for participants with tertiary education compared with those without, similar to previous findings by Scherer et al.^18^ Studies have shown that willingness to vaccinate^28^ or undergo cervical screening^29^ is often lower among people with less education, lower health literacy, and lower socioeconomic status. Other studies have reported that people with less education and lower health literacy have higher preferences for medication for cardiovascular disease^30^ and for more invasive surgery for papillary thyroid cancer.^31^ Whether or not preferences for more or less health care in people with less education and lower health literacy is a state or trait characteristic is an important question and needs more attention.

### Implications for Clinical Practice

Understanding personal factors, such as individuals’ diverse approaches to health care, might be beneficial in more effective communication and better involvement of individuals in health care choices, such as deciding to undergo screening or not. Some studies have suggested that the utility of the MMS lies in improving patient-clinician communication and the shared decision-making process in the clinical setting.^32^ Nurses, health coaches, and nonclinical members of a care team may be well placed to have in-depth conversations with patients regarding preferences for health care and could play a valuable role in administering and discussing MMS scores with patients considering PSA testing. Clinicians could use their patients’ orientation toward more or less health care (as measured by the MMS) as a useful starting point for tailored discussions, although some patients might have reservations about sharing their MMS score with their clinician.^32^ The challenge is to ensure that men who are considering having a PSA test have the opportunity to make an informed decision, which includes understanding the harms of screening, such as overdiagnosis.

More work is needed to examine communication strategies that help people direct attention to and consider information that goes against their usual preference for more or less care. Education and awareness of cognitive biases are important so that individuals can recognize their habitual thinking and attentional processes and try to reorient from their defaults toward more critical, evidence-based thinking. For example, cognitive (eg, using a consider-the-opposite procedure) or technological (eg, by providing graphical in addition to statistical information) debiasing strategies could encourage critical thinking and eliminate framing effects.^33^

The burden of decision-making should not lie solely with patients; health care practitioners should also be aware of their own perspectives and the possibility of cognitive biases influencing them. Future research could also explore clinician maximizing-minimizing orientation and associations with their approach to communication, screening, and diagnostic behavior. Changing clinician behavior (eg, only offering prostate cancer screening to patients for whom there is likely to be a strong benefit vs indiscriminate screening) and restructuring health care systems are key to reducing overdiagnosis from a systems perspective.^34^

### Limitations

This study has some limitations. Although the study used an online sample that is not fully representative of the male population of Australia aged 45 to 60 years, it allowed for a large and demographically diverse community sample, generally representative of the range of men who might be offered or are considering prostate cancer screening, and included a considerable proportion of men with lower educational attainment. This was not a longitudinal study, so we only measured MMS scores and knowledge, attitudes, and screening intentions once (ie, MMS was only measured at baseline). However, we collected a number of variables, which enabled a detailed analysis of the association of medical maximizing with key variables, with adjustment for confounders.

## Conclusions

This survey study is the first study, to our knowledge, to examine associations between people’s orientation toward more or less health care and knowledge about overdiagnosis and informed choice. A stronger preference for more health care was observed in participants without tertiary education and with inadequate health literacy. Preference for more health care was negatively associated with informed choice and understanding of overdiagnosis, even when controlling for education and health literacy. Understanding personal factors that influence diverse responses to health care information might be beneficial in contributing to more effective communication and better involving individuals in choices, such as deciding whether to undergo screening. Clinicians could use MMS scores as a useful starting point for tailored discussions with patients.

## Notes

Supplement. **eTable 1.** Included Measures **eTable 2.** Exploratory Subgroup Analysis by Previous PSA Test, Implemented as Interaction Terms in Regression Models **eFigure 1.** Estimated Risk of Positive Attitude Towards Screening, by MMS and Previous PSA **eFigure 2.** Estimated Risk of Positive Screening Intention, by MMS and Previous PSA
