# Supplementary material for: Preferences for More or Less Health Care and Association With Health Literacy of Men Eligible for Prostate-Specific Antigen Screening in Australia
Source: JAMA Netw Open. 2021 Oct 12;4(10):e2128380. doi: 10.1001/jamanetworkopen.2021.28380 (PMC8511975; doi:10.1001/jamanetworkopen.2021.28380)
Supplement: Supplement. — eTable 1. Included Measures eTable 2. Exploratory Subgroup Analysis by Previous PSA Test, Implemented as Interaction Terms in Regression Models eFigure 1. Estimated Risk of Positive Attitude Towards Screening, by MMS and Previous PSA eFigure 2. Estimated Risk of Positive Screening Intention, by MMS and Previous PSA [file jamanetwopen-e2128380-s001.pdf]

Supplemental Online Content

Pickles K, Scherer LD, Cvejic E, Hersch J, Barratt A, McCaffery KJ. Preferences for more or less health care and association with health literacy of men eligible for prostate-specific antigen screening in Australia. *JAMA Netw Open*. 2021;4(10):e2128380. doi:10.1001/jamanetworkopen.2021.28380

eTable 1. Included Measures

eTable 2. Exploratory Subgroup Analysis by Previous PSA Test, Implemented as Interaction Terms in Regression Models

eFigure 1. Estimated Risk of Positive Attitude Towards Screening, by MMS and Previous PSA

eFigure 2. Estimated Risk of Positive Screening Intention, by MMS and Previous PSA

This supplemental material has been provided by the authors to give readers additional information about their work.

© 2021 Pickles K et al. *JAMA Network Open.*

eTable 1. Included Measures

Item	Description and reference	Scoring & cut offs (where applicable)
**Explanatory variables –** Completed BEFORE viewing decision aid		
Age	What is your age (in years)?	Median (IQR) age (years)
Education	What is your highest level of education?<br><br>(University degree/Diploma or certificate/Trade apprenticeship/Higher school certificate or leaving certificate/School certificate or intermediate certificate/No school or other qualifications)	No tertiary education<br><br>Tertiary education
Private health insurance	Do you have private health insurance?<br><br>(Yes/No/Don't know)	
Health literacy	1 item, taken from [16], (1=Extremely confident to 5=Not at all confident)<br><br>How confident are you filling out medical forms by yourself?	Answers were dichotomised as "adequate" ("extremely" and "quite a bit") and "inadequate" ("somewhat", "a little bit" and "not at all").
Past PSA experience	a. Have you heard of the prostate-specific antigen (PSA) test? (Yes/No)<br>b. Have you ever had a prostate-specific antigen (PSA) test to screen for prostate cancer? (No/Yes in the last 12 months/Yes 1-2 years ago/Yes more than 2 years ago)<br>c. If yes, have you ever had an abnormal prostate-specific antigen (PSA) test? (Yes/No/Don't know)	
Medical Maximizer-Minimizer Scale (MMS)	10 items, taken from [2], (1=Strongly disagree to 7=Strongly agree)<br><br>a. It is important to treat disease even when it does not make a difference in survival<br>b. It is important to treat disease even when it does not make a difference in quality of life<br>c. Doing everything to fight illness is always the right choice<br>d. When it comes to health care, the only responsible thing to do is to actively seek medical care<br>e. If I have a health issue, my preference is to wait and see if the problem gets better on its own before going to the doctor (reverse coded 7 to 1)	Overall mean score (SD)<br><br>The mean score for each respondent is recorded, with a greater score indicating a preference towards seeking health care at a greater frequency than those scoring lower on the scale.

© 2021 Pickles K et al. *JAMA Network Open.*

	f. If I feel unhealthy, the first thing I do is to go to the doctor and get a prescription<br>g. I often suggest that friends and family see their doctor<br>h. When it comes to health care, watching and waiting is never an acceptable option<br>**i.** If I have a medical problem, my preference is to go straight to a doctor and ask his or her opinion<br>j. When it comes to medical treatment, more is usually better	
**Main outcomes –** Measured AFTER viewing decision aid		
Numerical knowledge	3 items, adapted from [27]:<br>"Imagine 1000 men who have PSA testing every 2 years from 50 to 69 years. Out of these 1000 men, over 20 years, about how many will…"<br>a. Still die from prostate cancer despite PSA testing?<br>b. Avoid dying from prostate cancer because of PSA testing?<br>c. Be overdiagnosed with prostate cancer because of PSA testing?	Mean (SD) total knowledge score<br>2 points were given for a correct answer, 1 point was given for an answer deemed reasonably close to correct.<br>Total knowledge score was calculated on a scale of 0 to 18 by adding up all conceptual and numerical knowledge questions.<br>**For informed choice**, the threshold to determine "adequate knowledge" was set a priori at 50% of total available knowledge marks, i.e. ≥9 points
Conceptual knowledge	4 items about prostate cancer, adapted from [27]:<br>a. Do you think a PSA screening test will find every prostate cancer? (Yes/No/Don't know)<br>b. Do all men with an abnormal PSA test result have prostate cancer? (Yes/No/Don't know)<br>c. Who do you think is more likely to die from prostate cancer? (Men who have PSA screening tests/Men who do not have PSA screening tests/Don't know)<br>d. Who do you think is more likely to be diagnosed with prostate cancer? (Men who have PSA screening tests/Men who do not have PSA screening tests/Don't know)	
Understanding of overdiagnosis	5 items about overdiagnosis, adapted from [27]:<br>a. Which of these statements best describes overdiagnosis? (Screening finds a cancer that would never have caused trouble/Screening finds an abnormality but extra tests show it is not cancer/Don't know)<br>b. All prostate cancers will eventually cause illness and death if they are not found and treated (True/False/Don't know)	

© 2021 Pickles K et al. *JAMA Network Open.*

	a. When screening finds cancer, doctors cannot reliably predict whether it will cause harm (True/False/Don't know)<br>b. Screening leads some men with a harmless cancer to get treatment they do not need (True/False/Don't know)<br>c. Screening finds harmless cancers more often than it prevents death from prostate cancer (True/False/Don't know)	
Previous awareness of overdiagnosis	1 item, taken from [11] (Yes/No/Don't know)<br><br>Have you seen or heard the term 'overdiagnosis' before today?	
Attitude toward screening, diagnosis and treatment	13 items, adapted from [28] (1=Strongly agree to 5=Strongly disagree)<br><br>a. I believe treatment will always help men live longer<br>b. If I had prostate cancer, I would prefer not to know<br>c. Having the PSA test would reassure me<br>d. Having a PSA test would do me more harm than good<br>e. Even though a prostate biopsy can miss cancer, I would still have one<br>f. If I had prostate cancer I believe I may live a better life without treatment<br>g. If I have a PSA test I believe it could lead to treatment that is not necessary<br>h. Having a PSA test would give me peace of mind<br>i. Having a PSA test would cause me to worry unnecessarily<br>j. I think the benefits of treatment for prostate cancer are more important than the side effects<br>k. I think all men my age should have a PSA test for prostate cancer<br>**l.** For me, having the PSA test is asking for trouble	Mean (SD) total attitudes score<br><br>Items scored 'Strongly agree' (2) to 'Strongly disagree' (-2).<br><br>Total scores could range from -24 to 24 with negative scores indicating a more negative attitude and positive scores indicating a more positive attitude (Positive score >0).<br><br>**For informed choice**, the threshold for a positive attitude was set at greater than zero.
Screening intention	1 item adapted from [29]:<br><br>At the moment, which of the following best describes your intentions about having a PSA screening test within the next 2-3 years?<br><br>(1=Definitely will have a PSA test to 5=Definitely will not have a PSA test)	**For informed choice**, item was dichotomised as "positive intention to screen" ("definitely will" and "likely to") and "negative intention to screen" ("unsure", "not likely" and "definitely will not")
Perceived risk	2 items adapted from [30]:<br><br>a. How likely do you think it is that you will develop prostate cancer in your lifetime? (1=No chance to 4=High chance)<br>b. Compared with the average man your age, how would you rate your chances	

© 2021 Pickles K et al. *JAMA Network Open.*

	of developing prostate cancer sometime in your life? (1=Much lower to 5=Much higher)	

Subgroup analysis by prior PSA test

To examine whether the association between MMS and outcomes differed by whether or not a participant had previously had a PSA test, the regression models were repeated (generalized linear models with Poisson distribution, log-link function and robust standard errors) including an interaction terms between MMS and previous PSA test. The interaction term p-values are shown in eTable 2.

© 2021 Pickles K et al. *JAMA Network Open.*

eTable 2. Exploratory Subgroup Analysis by Previous PSA Test, Implemented as Interaction Terms in Regression Models

**Outcome**	**MMS × Previous PSA interaction p-value**
Informed choice	.410
Positive attitude towards screening	.026
Previous awareness of overdiagnosis	.192
Correct understanding of overdiagnosis	.546
Correct conceptual knowledge	.910
Correct numerical knowledge	.521
Positive screening intention	<.001

There was evidence (p=.026) of an interaction between MMS and previous PSA for having a positive attitude towards screening. For individuals who had previously undergone PSA, the adjusted relative risk of having a positive attitude towards screening (per unit increase in MMS) was 1.14 (95%CI: 1.11, 1.18), compared to 1.21 (95%CI: 1.17, 1.25) per unit increase in MMS for individuals who had not previously undergone PSA. Predicted risks are shown in Figure S1.

© 2021 Pickles K et al. *JAMA Network Open.*

eFigure 1. Estimated Risk of Positive Attitude Towards Screening, by MMS and Previous PSA

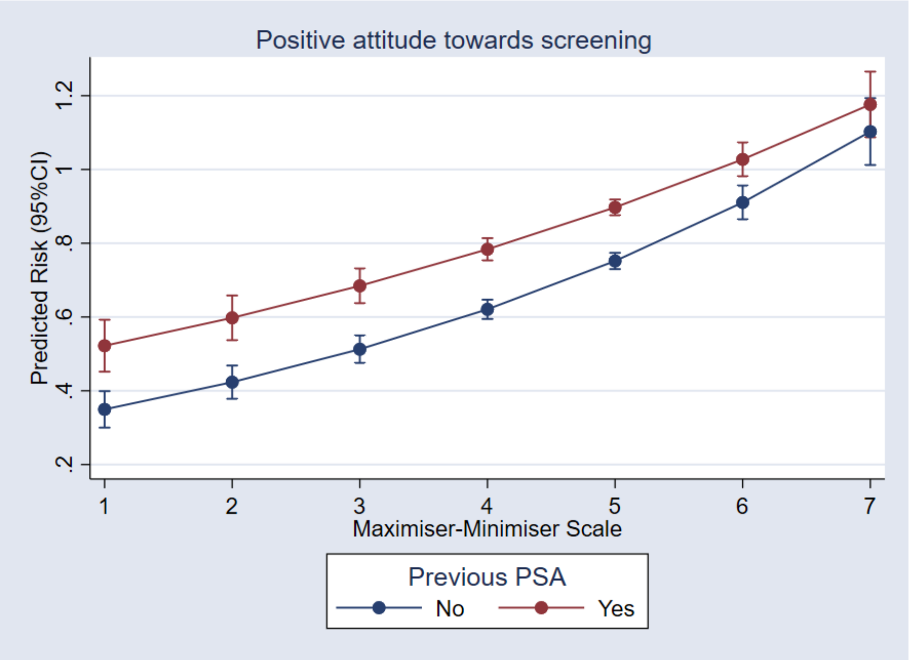

eFigure 1. Predicted risk of positive attitude towards screening, by MMS and previous PSA

There was very strong evidence (p<.001) of an interaction between MMS and previous PSA for positive screening intentions. For individuals who had previously undergone PSA, the adjusted relative risk of having positive screening intentions (per unit increase in MMS) was 1.11 (95%CI: 1.07, 1.15), compared to 1.32 (95%CI: 1.25, 1.40) per unit increase in MMS for individuals who had not previously undergone PSA. Predicted risks are shown in Figure S2.

© 2021 Pickles K et al. *JAMA Network Open.*

eFigure 2. Estimated Risk of Positive Screening Intention, by MMS and Previous PSA

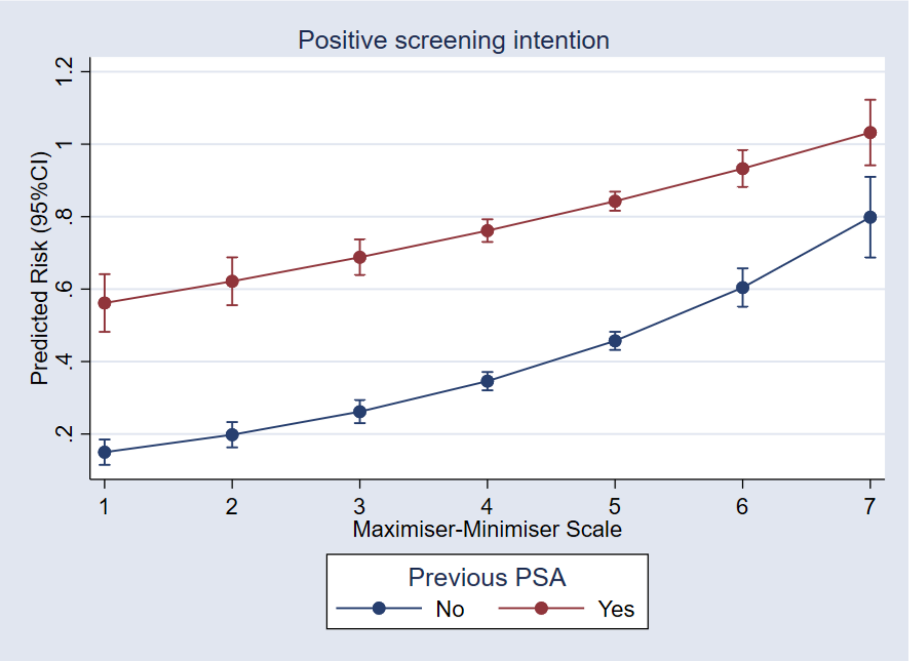

eFigure 2. Predicted risk of positive screening intention, by MMS and previous PSA

There was no evidence that the association of MMS was modified by previous PSA test for any other outcome.

© 2021 Pickles K et al. *JAMA Network Open.*
